# Frequent Occurrence of *Tomato Leaf Curl New Delhi Virus* in Cotton Leaf Curl Disease Affected Cotton in Pakistan

**DOI:** 10.1371/journal.pone.0155520

**Published:** 2016-05-23

**Authors:** Syed Shan-e-Ali Zaidi, Muhammad Shafiq, Imran Amin, Brian E. Scheffler, Jodi A. Scheffler, Rob W. Briddon, Shahid Mansoor

**Affiliations:** 1 Agricultural Biotechnology Division, National Institute for Biotechnology and Genetic Engineering, P O Box 577, Jhang Road, Faisalabad, Pakistan; 2 Pakistan Institute of Engineering and Applied Sciences (PIEAS), Islamabad, Pakistan; 3 Genomics and Bioinformatics Research Unit, 141 Experiment Station Rd., Stoneville, Mississippi, 38776, United States of America; 4 Crop Genetics Research Unit, United States Department of Agriculture-Agricultural Research Service (USDA-ARS), Stoneville, Mississippi, United States of America; Naval Research Laboratory, UNITED STATES

## Abstract

Cotton leaf curl disease (CLCuD) is the major biotic constraint to cotton production on the Indian subcontinent, and is caused by monopartite begomoviruses accompanied by a specific DNA satellite, Cotton leaf curl Multan betasatellite (CLCuMB). Since the breakdown of resistance against CLCuD in 2001/2002, only one virus, the “Burewala” strain of *Cotton leaf curl Kokhran virus* (CLCuKoV-Bur), and a recombinant form of CLCuMB have consistently been identified in cotton across the major cotton growing areas of Pakistan. Unusually a bipartite isolate of the begomovirus *Tomato leaf curl virus* was identified in CLCuD-affected cotton recently. In the study described here we isolated the bipartite begomovirus *Tomato leaf curl New Delhi virus* (ToLCNDV) from CLCuD-affected cotton. To assess the frequency and geographic occurrence of ToLCNDV in cotton, CLCuD-symptomatic cotton plants were collected from across the Punjab and Sindh provinces between 2013 and 2015. Analysis of the plants by diagnostic PCR showed the presence of CLCuKoV-Bur in all 31 plants examined and ToLCNDV in 20 of the samples. Additionally, a quantitative real-time PCR analysis of the levels of the two viruses in co-infected plants suggests that coinfection of ToLCNDV with the CLCuKoV-Bur/CLCuMB complex leads to an increase in the levels of CLCuMB, which encodes the major pathogenicity (symptom) determinant of the complex. The significance of these results are discussed.

## Introduction

Cotton is an important commodity and the export of cotton products is crucial for the economies of India and, especially, Pakistan. The cultivation of cotton across Pakistan and northwestern India is severely affected by cotton leaf curl disease (CLCuD) [1, 2]. The disease first came to prominence in the late 1980s near the city of Multan, Pakistan, and rapidly spread to almost all cotton growing areas of the country and into northwestern India. In the late 1990s cotton varieties obtained by conventional breeding and selection were introduced and rapidly restored production of cotton in Pakistan to the levels before the CLCuD epidemic. Unfortunately the disease appeared on all previously resistant varieties from 2001/2002 onwards. It was first observed near the town of Burewala Pakistan, indicating that the resistance had been broken [3]. This led to a second epidemic which rapidly spread to most areas of Pakistan and northwestern India.

Viruses of the genus *Begomovirus* are whitefly (*Bemisia tabaci*)-transmitted single-stranded (ss)DNA viruses that belong to family *Geminiviridae*. Begomoviruses occur in all the warmer parts of the World and infect only dicotyledonous plants [4]. In the Old World (OW) a small number of begomoviruses have been identified with genomes consisting of two components, known as DNA-A and DNA B. The majority of begomoviruses in the OW have a genome consisting of only a single component, homologous to the DNA-A component of bipartite viruses. The opposite is true in the New World where only one native monopartite begomovirus has been identified so far [5, 6]. The genomes of monopartite and DNA-A components of bipartite begomoviruses originating from the OW encode the coat protein (CP) and (A)V2 protein in the virion-sense orientation and the replication-associated protein (Rep; a rolling circle replication initiator protein), the replication enhancer protein (REn), the transcriptional activator protein (TrAP) and the C4 protein in the complementary-sense orientation [7]. DNA-B components encode the nuclear shuttle protein (NSP) and movement protein (MP) in the virion- and complementary-sense, respectively. The reading frames in the virion- and complementary-sense of begomovirus genomes/genomic components are separated by a non-coding (intergenic) region which contains cis-acting regulatory elements for gene expression, a predicted hairpin structure containing the conserved (among most geminiviruses) nonanucleotide sequence TAATATTAC as part of the loop and small repeated sequences, known as “iterons”, which are sequence specific binding sites for Rep. Together the iterons and hairpin form the origin of replication (*ori*) for virion-sense viral DNA replication. The sequence specific interaction between Rep and cognate iterons also ensures that the Rep of one virus will not initiate replication of the genome of a second virus. The DNA-A and DNA-B components of bipartite begomoviruses share a sequence, known as the common region (CR) that usually spans most of the intergenic region. The CR acts to maintain the integrity of the split genome ensuring that the DNA-A-encoded Rep can initiate replication of the virion strands for both components [8].

The majority of monopartite begomoviruses are associated with additional small ssDNA molecules known as betasatellites and alphasatellites [9]. Betasatellites (previously known as DNA-β) have so far only been identified in the OW. They are half the size of begomovirus components (∼1350 nt) and encode a single gene on the complementary-sense strand that codes for an ∼118 amino acid protein known as βC1. Betasatellites mayincrease the accumulation of their helper begomoviruses, as well as enhance symptoms in some host plants [10, 11]. This is likely due to βC1 having suppressor of RNA interference activity [12, 13].

The alphasatellites (previouslyknownasDNA-1; [14]) are not strict satellites, since they are capable of autonomous-replication in permissive plant cells. They are dependent on their helper begomoviruses for movement within plants and insect transmission between plants [15, 16]. Although widespread in the OW, alphasatellites have also been identified in the NW in association with bipartite begomoviruses, in the absence of betasatellites [17, 18]. Recently an alphasatellite and a betasatellite were shown in association with a mastrevirus (genus *Mastrevirus*, family *Geminiviridae*) [19].

CLCuD in Pakistan and northwestern India during the 1990s was shown to be associated with at least four monopartite begomoviruses including *Cotton leaf curl Multan virus* (CLCuMuV), *Cotton leaf curl Alabad virus* (CLCuAV), *Cotton leaf curl Kokhran virus* (CLCuKoV) and *Papaya leaf curl virus* (PaLCuV) [20, 21]. Of these only CLCuMuV, CLCuKoV and PaLCuV have been shown experimentally to cause CLCuD in cotton in the presence of a distinct betasatellite—Cotton leaf curl Multan betasatellite (CLCuMB)[10, 21]. After the breakdown of resistance in 2001–2002, CLCuD across the Punjab province of Pakistan was shown to be associated with a single monopartite begomovirus; the “Burewala” strain of CLCuKoV (CLCuKoV-Bu; previously called *Cotton leaf curl Burewala virus*). CLCuKoV-Bu is a recombinant virus with some sequences derived from CLCuMuV [22]. Unusually CLCuKoV-Bu associated with resistance breaking lacked one of the usual complement of genes [22, 23] and was associated with a recombinant form of CLCuMB (CLCuMB^Bur^) with some sequence derived from another betasatellite [24].

*Tomato leaf curl New Delhi virus* (ToLCNDV) is an unusual begomovirus. It is one of very few bipartite begomoviruses in the OW and has been reported from a large number of different plants, including members of the *Solanaceae*, *Cucurbitaceae* and *Malvaceae* [24–28]. The virus is also unusual in sharing its DNA-B component with a number of other bipartite begomoviruses, including *Pepper leaf curl Bangladesh virus*[29], *Tomato leaf curl Palampur virus*[30], *Bhendi yellow vein mosaic virus* [28] and with *Tomato leaf curl virus*, a begomovirus for which some isolates are monopartite [31].

Recently we have identified an isolate of ToLCV with a DNA-B in CLCuD-affected cotton plants originating from the Punjab; the first time a bipartite begomovirus was identified in cotton on the Indian subcontinent [32]. The study here reports the occurrence of the bipartite ToLCNDV in CLCuD-symptomatic cotton for the first time. Additionally the widespread presence of ToLCNDV in CLCuD-affected cotton over a wide area of the cotton growing regions of the Punjab and Sindh provinces of Pakistan is shown. The effects of the presence of ToLCNDV on the levels of DNA-B, virus and satellites were investigated by quantitative PCR. The implications of these findings are discussed.

## Materials and Methods

### Origins of plant materials and DNA extraction

**Fig 1:**
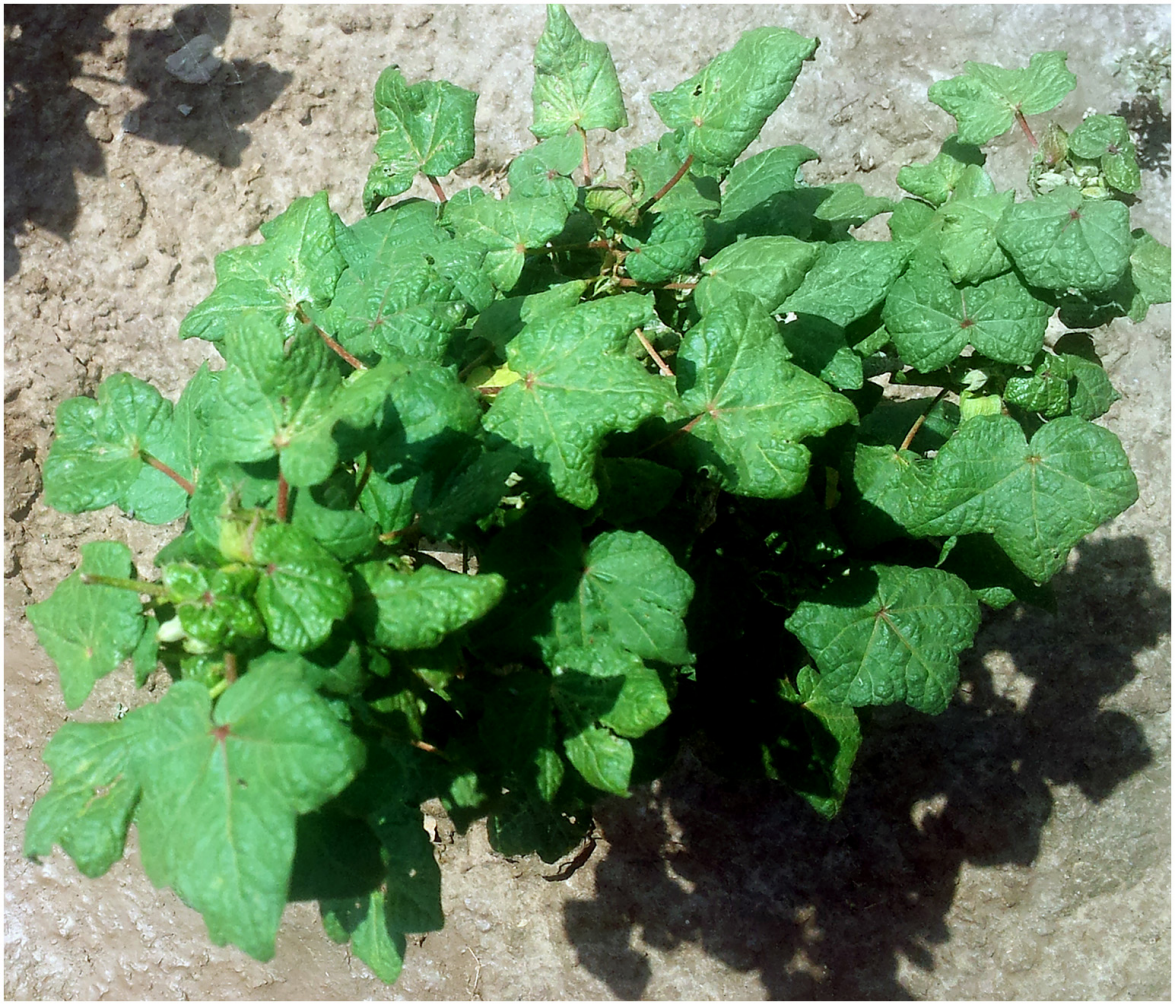
Cotton (*Gossypium hirsutum*) plant exhibiting typical symptoms of cotton leaf curl disease.

Leaf samples from cotton plants with symptoms typical of CLCuD, consisting of leaf curling, vein thickening, vein yellowing, enations and stunted growth (Fig 1), and from apparently healthy plants,were collected from areas of Punjab and Sindh provinces of Pakistan between2013 and 2015 (Table 1). DNA was extracted from samples using a cetyltrimethyl ammonium bromide (CTAB) method [33]. DNA was quantified using a Nanodrop spectrophotometer (Thermo Fisher Scientific, Waltham, MA USA).

**Table 1:** Origins offield collected cotton samples and presence of viruses.

Location		Isolate	GPS coordinates		Virus*	
Province	District		North	East	ToLCNDV	CLCuKoV
Punjab	Bahawalpur	C140	29.59636	72.83348	-	✓
Punjab	Bahawalpur	C150	29.56129	72.74316	-	✓
Punjab	Bahawalpur	K201	29.10163	71.7427	✓	✓
Punjab	Bahawalpur	K202	29.10163	71.7427	✓	✓
Punjab	Bahawalpur	K203	29.20806	71.75604	✓	✓
Punjab	Bahawalpur	K204	29.20806	71.75604	✓	✓
Punjab	Bahawalpur	K206	29.17652	71.86785	-	✓
Punjab	Bahawalpur	K207	29.17652	71.86785	✓	✓
Punjab	Bhakkar	D11	31.43663	71.52154	-	✓
Punjab	Bhakkar	D26	31.48838	71.26929	-	✓
Punjab	Faisalabad	C31	31.35432	73.30094	-	✓
Punjab	Khanewal	H38	30.40681	72.12185	-	✓
Punjab	Khanewal	H41	30.35511	72.08303	-	✓
Punjab	Khanewal	H42	30.35511	72.08303	-	✓
Punjab	Toba Tek Singh	H1	31.31402	72.79922	-	✓
Punjab	Toba Tek Singh	H11	31.05338	72.58824	-	✓
Punjab	Toba Tek Singh	H20	30.87548	72.53648	✓	✓
Punjab	Toba Tek Singh	H9	31.05338	72.58824	✓	✓
Punjab	Faisalabad	NIAB1	31.3982	73.0339	✓	✓
Punjab	Faisalabad	NIAB2	31.3982	73.0339	✓	✓
Sindh	Ghotki	K123	28.10269	69.71552	✓	✓
Sindh	Nawabshah	SS23	26.1255	68.2804	✓	✓
Sindh	Nawabshah	SS32	26.1255	68.2804	✓	✓
Sindh	Nawabshah	SS33	26.1255	68.2804	✓	✓
Sindh	Nawabshah	SS34	26.1255	68.2804	✓	✓
Sindh	Nawabshah	SS35	26.1255	68.2804	✓	✓
Sindh	Tando Allahyar	SS1	25.4799	68.7900	✓	✓
Sindh	Mirpur Khas	SS10	25.5101	68.9301	✓	✓
Sindh	Mirpur Khas	SS11	25.5101	68.9301	✓	✓
Sindh	Hayderabad	SS16	25.4997	68.4196	✓	✓
Sindh	Matiari	SS20	25.9299	68.3800	✓	✓

*The presence of either *Tomato leaf curl New Delhi virus* (ToLCNDV) or *Cotton leaf curl Kokhran virus* (CLCuKoV) was determined by diagnostic PCR with ToLCNDV DNA-B specific primers or with the CLCuKoV specific primers described by Shuja *et al*.[38].

### Ethics statement

The National Institute for Biotechnology and Genetic Engineering (NIBGE) is a public sector institute, the employees of which areauthorized to visit farmer’s fields and collect plant samples. However, before going to any private field, verbal permission was sought from the owner of the field. The field studies did not involve endangered or protected species.

### Virus Amplification, Cloning and Sequencing

Rolling circle amplification (RCA) using phi 29 DNA polymerase (ThermoFisher Scientific, Waltham, MA USA) was used to amplify all circular DNA molecules in 31cotton samples [34]. The DNA-A and DNA-B components of ToLCNDV were amplified by polymerase chain reaction (PCR)with RCA-enriched nucleic acid samples as the template and using the specific primer pairsToLCNDV_A1_Forward (GATATCATCATTTCAACGCCCGCATCGAA)/ToLCNDV_A2_Reverse (GATATCTGCTGGTCGCTTCGCCATAGTTC), ToLCNDV_A3_Forward (GAGCTCGTGCAGTTGTCCCCATTGCCCGCGTCAC)/ToLCNDV_A4_Reverse(GAGCTCCATAGGGGCTGTCGAAGTTG) for DNA-A and ToLCNDV_B1_Forward (AAGCTTCTGCTCGAACATGGACGGAAATGAC)/ToLCNDV_B2_Reverse (AAGCTTAGCCAGTTGAGGAATAGATGCATG), ToLCNDV_B3_Forward (GGTACCCGTAACGATCTTGAACTATGTCCC)/ToLCNDV_B4_Reverse (GGTACCCTATCTGGCTATAGGTCCGAACG) for DNA-B. The degenerate primers BegomoF and BegomoR were used to PCR amplify the genomes of monopartite and/or DNA-A components of bipartite begomoviruses [35]. Betasatellites and alphasatellites were PCR-amplified using universal primers [36, 37]. The primers described by Shuja et al. [38] were used to specifically detect CLCuKoV.

PCR reactions for cloning used RCA product as the template. Amplification products of ~2.8 kb, for virus, DNA-A or DNA-B, and ~1.4 kb for alphasatellite and betasatellite, were cloned into a T/A cloning vector (pTZ57R/T; ThermoFisher Scientific, Waltham, MA USA). From each isolate 3–5 clones were sequenced. Sequences were determined by dideoxy nucleotide chain termination sequencing on an Applied Biosystems 3730XL DNA sequencer and were assembled and manipulated using the Lasergene package of sequence analysis software (DNAStar Inc., Madison, WI, USA). The MUSCLE option of the Sequence Demarcation Tool [39] was used to analyze the sequenced clones for the identification of distinct geminivirus species according to revised taxonomy of begomoviruses based on pairwise sequence comparisons [40]. Sequences were aligned using CLUSTAL W [41] implemented in MEGA6 [42]. Phylogenetic analyses were conducted on aligned sequences using the neighbor-joining and bootstrap options of CLUSTAL X and visualized in TreeView [43].

### Quantitative real-time PCR

Real-time PCR reactions consisted of a total volume of 25 μL with 12.5 μL of SYBER Green Super Mix (Thermo Fisher Scientific, Waltham, MA USA), 0.25 μL of each primer (0.1 μM each), 2.5 μL of DNA (25 ng), and 9.5 μL water. The cycling conditions were an initial 94°C for 10 min, followed by 40 cycles of 30 seconds (s) at 94°C, 30 s at 57°C, 30 s at 72°C, followed by melt curve analyses. Reactions were performed in a 96 well microtitre plate format using an iQ5 thermal cycler (Bio-Rad, Hercules, CA USA). The 18S ribosomal RNA gene was used as a reference gene to normalize DNA levels in samples. Each sample was run in triplicate.

Standard curves for absolute quantification were obtained from five sets of tenfold serial dilutions (starting from 20 ng/μL) of a plasmid containing the cloned full-length CLCuMB (AJ298903; [10]) ToLCNDV DNA-A (U150150) and DNA-B (U15017; [44]) were dissolved in 20 ng/μL of genomic DNA extracted independently from healthy cotton plants, to obtain a range from 20 ng/μL to 0.002 ng/μL of each component. The primers used in the quantitative PCR analyses were DNA_A_qPCR_Forward (CCTTTAATCATGACTGGCTT)/DNA_A_qPCR_Reverse (CATTTCCATCCGAACATTC) for begomovirus genome or DNA-A component, DNA_B_qPCR_Forward (GCCCATGATTCGTTCGGAC)/DNA_B_qPCR_Reverse (CACGTGGTACTGGAATATCGCA) for DNA-B and Betasatellite_qPCR_Forward (GATTTGACTTATATTGGGCCAATTTAAT)/Betasatellite_qPCR_Reverse (GATACTATCCACAAAGTCACCATCGCTAAT) for betasatellites.

## Results

### Identification of ToLCNDV in CLCuD affected cotton

Clones of ~ 2.8 kb and ~1.4kb were obtained from 10 cotton plants with CLCuD symptoms (Tables 2, 3 and 4). The virus and satellite sequences obtained were analyzed for the presence of potential genes. The analysis showed that the arrangement of genes for 7 clones was typical of either monopartite begomoviruses or the DNA-A component of bipartite begomoviruses (Table 2).

**Table 2:** Origins and features of monopartitebegomovirus and bipartite begomovirus DNA-A component clones obtained.

Clone	Virus/virus component	GPS coordinates	Location (province/district)	Accession no.	Size (nt)	Coding sequences [coordinates/no. of amino acids]					
						CP	V2	Rep	TrAP	REn	C4
SAZ21*	CLCuKoV-Bur	29.17652N/71.86785E	Punjab/Bahawalpur	LN845931	2758	291-1061/256	131-487/118	1504-2595/363	1500-1607/35	1058-1462/134	2241-2681/146
SAZ22*	CLCuKoV-Bur	29.17652N/71.86785E	Punjab/Bahawalpur	LN845932	2758	291-1061/256	131-487/118	1504-2595/363	1500-1607/35	1058-1462/134	2241-2681/146
SAZ26*	CLCuKoV-Bur	29.17652N/71.86785E	Punjab/Bahawalpur	LN713267	2758	291-1061/256	131-487/118	1504-2595/363	1500-1607/35	1058-1462/134	2241-2681/146
SAZ27*	CLCuKoV-Bur	29.17652N/71.86785E	Punjab/Bahawalpur	LN713268	2758	291-1061/256	131-487/118	1504-2595/363	1500-1607/35	1058-1462/134	2241-2681/146
SAZ37*	CLCuKoV-Bur	29.17652N/71.86785E	Punjab/Bahawalpur	LN713271	2759	292-1062/256	132-488/118	1505-2596/363	1501-1608/35	1059-1463/134	2242-2682/146
SAZ33*	CLCuKoV-Bur	29.10163N/71.7427E	Punjab/Bahawalpur	LN845933	2759	292-1062/256	132-488/118	1505-2596/363	1501-1608/35	1059-1463/134	2242-2682/146
SAZ34^@^	ToLCNDV DNA-A	29.17652N/1.86785E	Punjab/Bahawalpur	LN845962	2738	280-1050/256	120-458/112	1499-2584/361	1177-1596/139	1047-1457/136	2251-2427/58

* Clones produced using primers BegomoF and BegomoR

^@^ Clone produced using primers ToLCNDV_A3 and ToLCNDV_A4

**Table 3:** Origins and features of bipartite begomovirus DNA-B component clones obtained.

Clone	Virus component	Location (province/district)	Accession no.	Size	Coding sequence [coordinates/no. of amino acids]	
					MP	NSP
SAZ 28	ToLCNDV DNA-B	Punjab/Bahawalpur	LN713269	2695	1305-2150/281	440-1126/228
SAZ 30	ToLCNDV DNA-B	Punjab/Bahawalpur	LN845955	2693	1305-2150/281	440-1246/268
SAZ 31	ToLCNDV DNA-B	Punjab/Bahawalpur	LN713270	2692	1304-2149/281	441-1247/268
SAZ 35	ToLCNDV DNA-B	Punjab/Bahawalpur	LN845956	2692	1304-2149/281	441-1247/268
SAZ 68	ToLCNDV DNA-B	Punjab/Bahawalpur	HG983285	2688	1301-2146/281	438-1244/268
SAZ 181	ToLCNDV DNA-B	Punjab/Bahawalpur	LN845934	2687	1301-2146/281	438-1244/268
SAZ 235	ToLCNDV DNA-B	Punjab/Bahawalpur	LN854628	2694	1304-2149/281	440-1126/228

**Table 4:** Origins and features of alphasatellite and betasatellite clones obtained.

Clone	Satellite	Location (province/district)	Accession no.	Size(nt)	Coding sequence [coordinates/no. of amino acids]	
					βC1	Rep
SAZ 4	CLCuMB	Punjab/Bahawalpur	LN845926	1370	195-551/118	-
SAZ 6	CLCuMB	Punjab/Bahawalpur	HG934394	1369	195-551/118	-
SAZ 13	CLCuMB	Punjab/Khanewal	LN845927	1350	195-551/118	-
SAZ 14	CLCuMB	Punjab/Khanewal	LN845928	1350	195-551/118	-
SAZ 15	CLCuMB	Punjab/Khanewal	HG934395	1350	195-551/118	-
SAZ 24	CLCuMB	Punjab/Khanewal	LN845930	1350	195-551/118	-
SAZ 25	CLCuMB	Punjab/Khanewal	HG934396	1350	195-551/118	-
SAZ 251	CLCuMB	Punjab/Faisalabad	LN867444	1357	195-551/118	-
SAZ 253	CLCuMB	Punjab/Faisalabad	LN867445	1370	195-551/118	-
SAZ 254	CLCuMB	Punjab/Faisalabad	LN867446	1370	195-551/118	-
SAZ 255	CLCuMB	Punjab/Faisalabad	LN867447	1370	195-551/118	-
SAZ 257	CLCuMB	Punjab/Faisalabad	LN867448	1351	195-551/118	-
SAZ 261	CLCuMB	Punjab/Faisalabad	LN867449	1352	195-551/118	-
SAZ 265	CLCuMB	Punjab/Faisalabad	LN867450	1370	195-551/118	-
SAZ 1	CLCuMA	Punjab/Khanewal	LN845921	1365	-	77-1024/315
SAZ 2	CLCuMA	Punjab/Khanewal	HG934390	1366	-	77-1024/315
SAZ 3	CLCuMA	Punjab/Khanewal	LN845925	1366	-	77-1024/315
SAZ 10	CLCuMA	Punjab/Bahawalpur	LN845922	1377	-	77-1024/315
SAZ 11	CLCuMA	Punjab/Bahawalpur	LN845923	1377	-	77-1024/315
SAZ 12	CLCuMA	Punjab/Bahawalpur	HG934391	1377	-	77-1024/315
SAZ 17	CLCuMA	Punjab/Bahawalpur	HG934392	1365	-	77-1024/315
SAZ 19	CLCuMA	Punjab/Bahawalpur	LN845924	1366	-	77-1024/315
SAZ 20	CLCuMA	Punjab/Bahawalpur	HG934393	1365	-	77-1024/315

**Fig 2:**
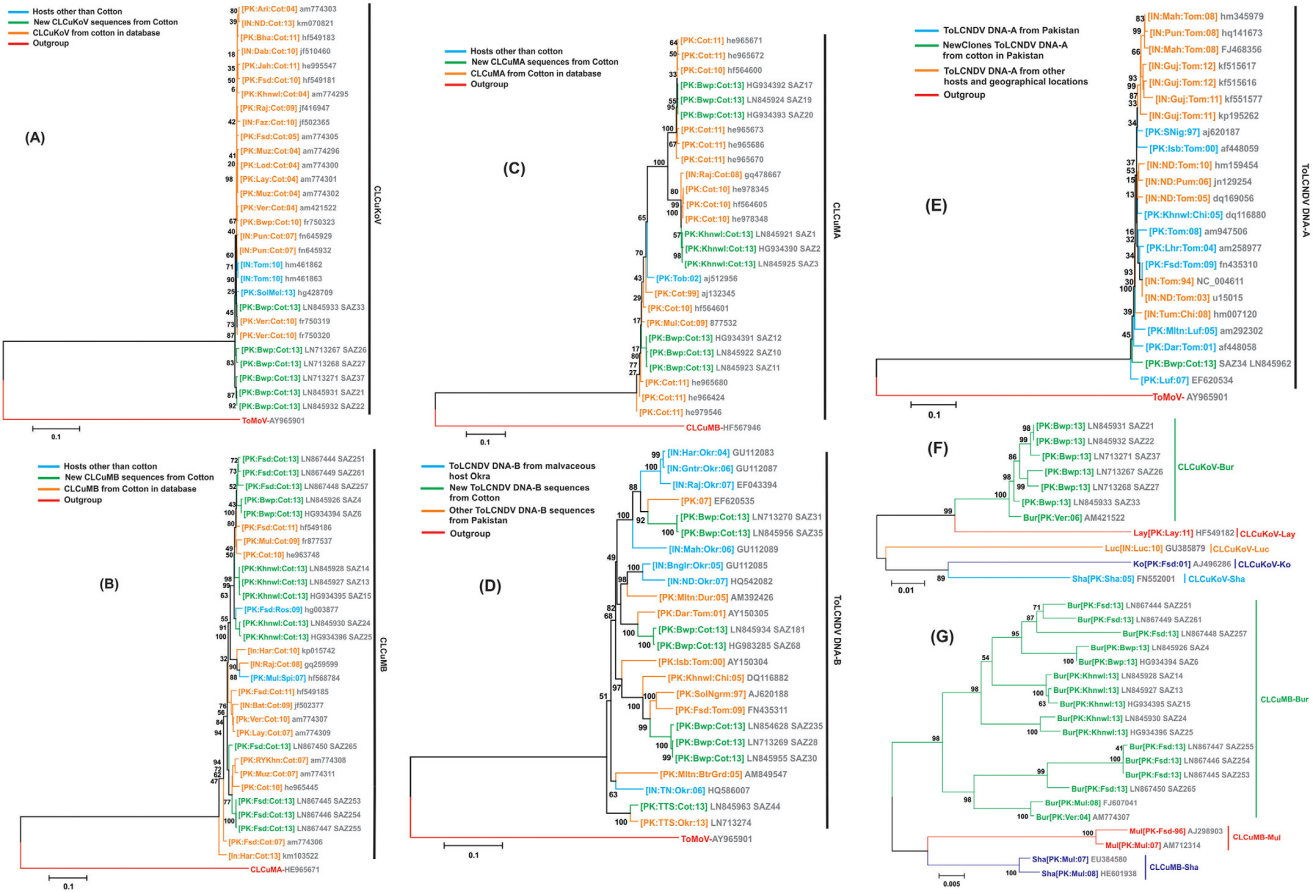
Phylogenetic analysis of the sequences of begomoviruses, and satellites obtained in the study. Trees were constructed from alignments of the sequences of (A and F) *Cotton leaf curl Kokhran virus* (CLCuKoV), (B and G) Cotton leaf curl Multan betasatellite (CLCuMB), (C) Cotton leaf curl Multan alphasatellite (CLCuMA), (D) *Tomato leaf curl New Delhi virus* (ToLCNDV) DNA-B and (E) ToLCNDV DNA-A using the Neighbor-Joining method. Isolates in green were obtained in the study described here. The numbers at nodes represent percentage bootstrap scores (1000 replicates). The strain descriptors (in square brackets) in each case give country, location, host and year of sampling. For each isolate the database accession number is given. The trees were arbitrarily rooted on the sequence of *Tomato mottle virus* (ToMoV) (A, D and E), CLCuMA (B) and CLCuMB (C) as outgroup. The strains of CLCuKoV given in panel F are Burewala (Bur), Layyah (Lay), Lucknow (Luc), Kokhran (Kok) and Shadadpur (Sha). The strains of CLCuMB given in panel G are given as Burewala (Bur), Multan (Mul) and Shadadpur (Sha).

A closer analysis indicated that 6 of these clones have a truncated TrAP gene, with a potential coding capacity of 35 amino acids, whereas one clone (SAZ34) encoded a putatively full-length TrAP predicted to be of 136 amino acids (Table 2). Mutation of the TrAP gene is typical of CLCuKoV-Bur isolates associated with resistance breaking [22]. The six clones obtained here show 98–100% nucleotide sequence identity with CLCuKoV isolates available in the databases. In phylogenetic analyses the sequences obtained here show low branch lengths to previously characterized CLCuKoV isolates (Fig 2, panel A) and segregate with CLCuKoV-Bur isolates (Fig 2, panel F). This confirms that the sequences obtained here are isolates of CLCuKoV-Bur.

Analysis of the sequence of clone SAZ34 showed it to have 94–96% sequence identity with the sequences of ToLCNDV component DNA-A available in the databases. A phylogenetic analysis showed the sequence to have low branch lengths to the DNA-A components of previously characterized ToLCNDV isolates (Fig 2, panel E). This confirmed that ToLCNDV was present in the cotton plant analysed.

The remaining seven ~2.8kb clones had an arrangement of genes typical of the DNA-B component of bipartite begomoviruses, consisting of one gene encoded in each orientation (Table 3). These sequences showed 82–92% nucleotide sequence identity to the sequences of the DNA-B components of ToLCNDV available in the databases. In phylogenetic analysis the six sequences obtained here had short branch lengths to the sequences of the DNA-B components of ToLCNDV obtained from the databases (Fig 2, panel D). This confirms that the clones are isolates of the DNA-B component of ToLCNDV.

### An alphasatellite is associated with cotton leaf curl disease

A total of 23 ~1.4kb clones were obtained from eight CLCuD-affected cotton plants. Analysis of the sequences showed them to encode either one large (~950bp) gene in the virion-sense, typical of alphasatellites, or a small (~350bp) gene in the complementary-sense, typical of betasatellites (Table 4). The 9 presumed alphasatellite clones showed 89–99% nucleotide sequence identity to isolates of Cotton leaf curl Multan alphasatellite (CLCuMA) available in the databases. A phylogenetic analysis also showed the 9 sequences to group with low branch lengths with the sequences of CLCuMA available in the databases (Fig 2, panel C). This confirms that the alphasatellites isolated from cotton here are isolates of CLCuMA.

The 14 presumed betasatellite clones showed 91–99% nucleotide sequence identity to Cotton leaf curl Multan betasatellite (CLCuMB) sequences available in the databases. In a phylogenetic analysis the new sequences group with short branch lengths with the sequences of earlier reported CLCuMB isolates (Fig 2, panel B). Additionally the CLCuMB sequences obtained here segregated with isolates of the “Burewala” strain of CLCuMB (CLCuMB^Bur^) rather than the “Multan” or “Shadadpur” strains (Fig 2, panel G). This shows that the cotton plants examined were infected with CLCuMB^Bur^, which is associated with resistance breaking [45].

### Geographic incidence of co-infection of cotton with ToLCNDV and CLCuKoV-Bur

**Fig 3:**
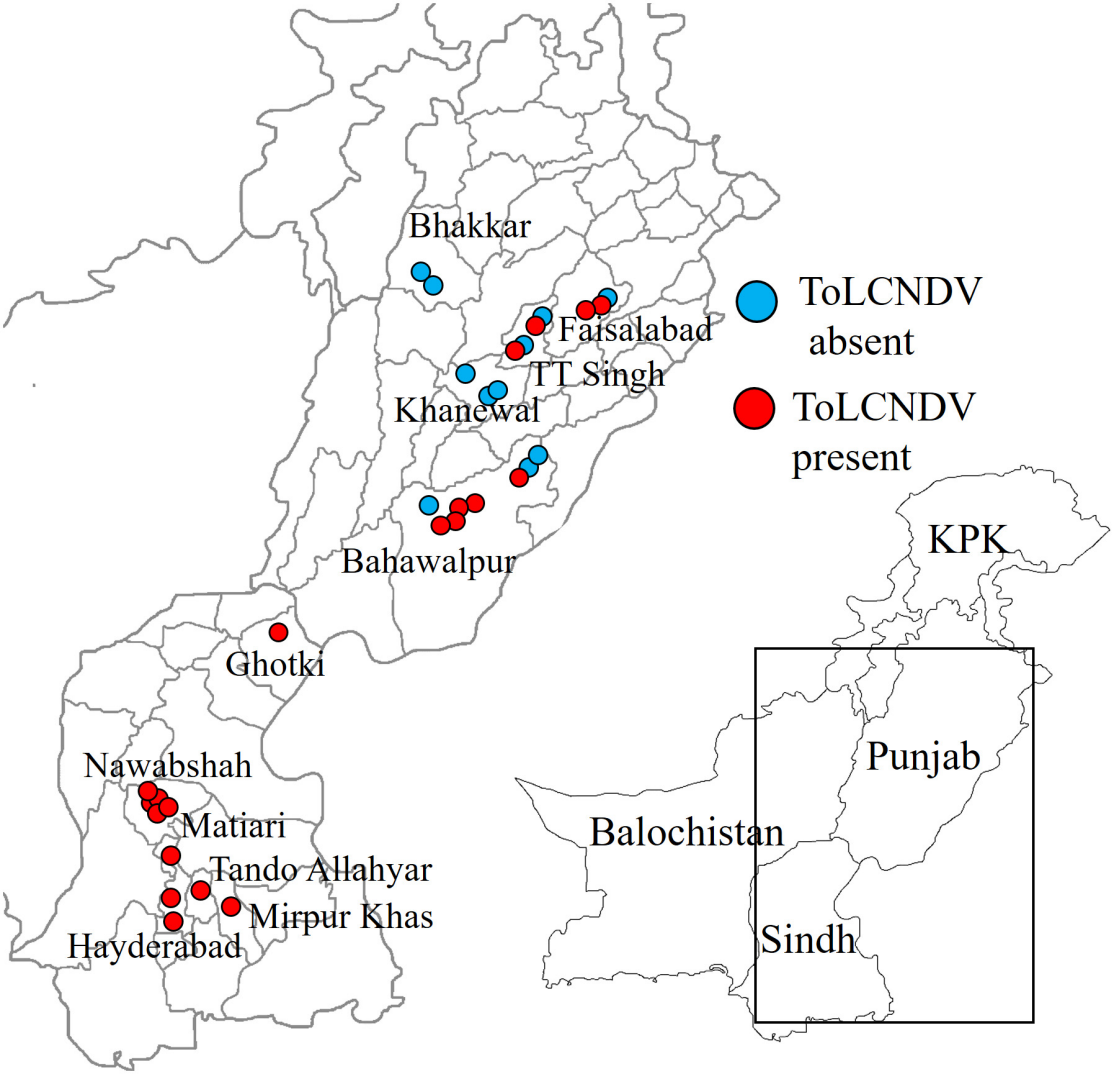
Distribution of samples shown to harbour coinfection of CLCuKoV-Bur and ToLCNDV across Pakistan. The map shows the origin of collected cotton plant samples with CLCuD symptoms. Plants shown by diagnostic PCR to contain both CLCuKoV-Bur and ToLCNDV are shown as red dots, whereas plants shown only to contain CLCuKoV-Bur are shown as blue dots. The map depicting putative boundaries is drawn by the author and provided here for illustrative purpose only.

The incidence and area over which coinfection of cotton with ToLCNDV and the CLCuKoV-Bur/CLCuMB complex was investigated by diagnostic PCR with primers specific for ToLCNDVDNA-B and CLCuKoV-Bur on DNA samples extracted from CLCuD symptomatic cotton originating from across the Punjab and northern Sindh provinces of Pakistan (Fig 3; Table 1.). Of the 31 samples examined, 20showed the presence of both viruses. The plants harbouring both viruses originated from geographically widespread areas across the Punjab and Sindh. These results indicate that ToLCNDV is widespread in cotton in Pakistan.

### Quantitative PCR analysis of CLCuKoV-Bur/CLCuMB and ToLCNDV coinfected cotton plants

**Fig 4:**
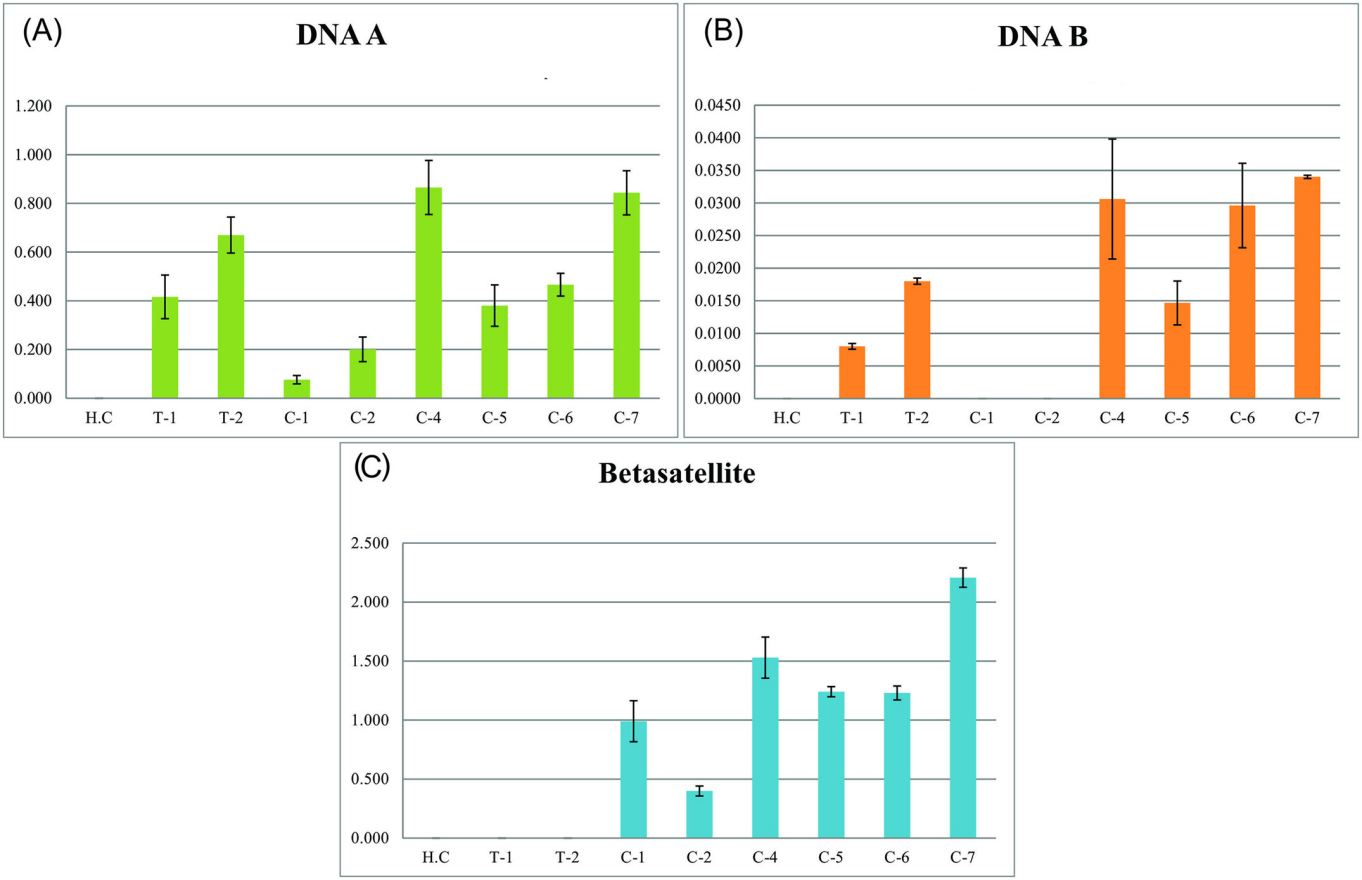
Quantitative real-time PCR analysis of coinfected cotton plants. Graphs show the determined titers of (A) virus (*Tomato leaf curl New Delhi virus* [ToLCNDV] DNA-A and *Cotton leaf curl Kokhran virus*-Burewala CLCuKoV-Bur), (B) ToLCNDV DNA-B and (C) Cotton leaf curl Multan betasatellite (CLCuMB). The DNA samples used in the PCR reactions were extracted from a healthy cotton plant (H.C), tomato plants infected with ToLCNDV (T1 and T2), field collected cotton plants with severe cotton leaf curl disease symptoms either infected with only CLCuKoV-Bur/CLCuMB (C-1 and C-2) or coinfected with CLCuKoV-Bur/CLCuMB and ToLCNDV (C-4 to C-7). The titer of each component is given in ng/μg of genomic DNA on the y-axis and is the mean of three replications. The error bars are the divergence from mean quantified value.

The results of a quantitative PCR analysis of the levels of virus (the primers used amplify both ToLCNDVDNA-A and CLCuKoV-Bur), ToLCNDVDNA-B and CLCuMB in CLCuD symptomatic cotton plants infected with only the CLCuKoV-Bur/CLCuMB complex or co-infected with CLCuKoV-Bur/CLCuMB and ToLCNDV are shown in Fig 4. The analysis indicates that in coinfected cotton plants the levels of CLCuMB are significantly higher than in cotton plants infected with only CLCuKoV-Bur/CLCuMB.

## Discussion

Begomovirus disease complexes are evolving rapidly by recombination, component capture and mutation to expand their host range and overcome sources of resistance. The resistance breaking begomovirus-betasatellite complex causing CLCuD evolved by recombination and mutation[22, 23, 45]. The susceptibility of previously resistant/tolerant cotton lines to the disease prompted aninvestigation into possiblechanges in the disease complex since resistance breaking. The results obtained here are consistent with the present belief that CLCuD in resistant cotton varieties across Pakistan and northwestern India is caused by CLCuKoV-Bur and CLCuMB^Bur^. Recently CLCuKoV-Bur and CLCuMB^Mul^ have been shown experimentally to be able to cause CLCuD in cotton [46]. However, this study did not investigate whether this combination of virus and betasatellite could break resistance in cotton.

In many of the cotton plants examined here identified the alphasatellite CLCuMA was identified. This indicates that, as was the case before resistance breaking, the virus causing CLCuD is associated with an alphasatellite. The study of Amrao et al. [22] which first identified CLCuKoV-Bur, reported that there was no evidence for the presence of an alphasatellite. The study here is thus the first to report an alphasatellite with the resistance breaking complex. The precise functions of alphasatellites remain unclear, although evidence has been provided to show that alphasatellites may encode a suppressor of gene silencing which overcome host resistance based on small RNAs [47].

The most surprising finding of the study presented here was the presence of the bipartite begomovirus ToLCNDV in cotton affected by CLCuD. A number of other geminiviruses have been identified in cotton including the mastrevirus *Chickpea chlorotic dwarf virus* [48], ToLCV [32] and *Okra enation leaf curl virus* [49]. However, these viruses were only identified across a limited area and in a few plants. ToLCNDV, in contrast, was identified in cotton across a wide area of Pakistan, suggesting that it is more than just a fleeting infection.

The quantitative PCR analysis suggests that in cotton there is a synergistic interaction between CLCuKoV-Bur/CLCuMB^Bur^ complex and ToLCNDV which leads to an increase in the amount of CLCuMB^Bur^ present in coinfected plants. Betasatellites encode a dominant symptom determinant [50, 51] and the βC1 gene of CLCuMB alone has been shown to induce symptoms typical of CLCuD in tobacco [52]. Any increase in betasatellite levels with a concomitant increase in βC1 gene is thus undesirable.

The nature of a possible synergistic interaction between the CLCuKoV-Bur/CLCuMB^Bur^ complex and ToLCNDV is unclear. The DNA-A component of ToLCNDV, in the absence of the DNA-B, has been shown to be able to support the replication of CLCuMB in cotton and, at least transiently, induce typical CLCuD symptoms [53]. A study of the interaction of ToLCNDV with CLCuMB in tomato and *Nicotiana benthamiana* showed the presence of CLCuMB to enhance the viral DNA levels but the presence of DNA-B depressed CLCuMB levels [54]. Nevertheless, the increase in betasatellite and possibly virus levels in coinfected cotton may be due to the movement functions encoded by the DNA-B component of ToLCNDV allowing the infection to spread to tissue which it normally does not reach [55].

CLCuD is a major constraint to cotton production in Pakistan and India. At this time there are no commercially available cotton varieties with resistance to the disease. The appearance of a form of the virus-complex causing the disease with potentially enhanced pathogenicity is thus not good news. Further studies will be needed to monitor the situation and see whether the coinfection persists and precisely what the effects are on the yield of cotton. Additionally, any efforts towards developing resistance to the disease, either by conventional or non-conventional means, would be wise to take into account the possibility of a more complex situation becoming important in cotton in the future.
